# Supplementary material for: Synergistic Antimicrobial Effects of Phage vB_AbaSi_W9 and Antibiotics against Acinetobacter baumannii Infection
Source: Antibiotics (Basel). 2024 Jul 22;13(7):680. doi: 10.3390/antibiotics13070680 (PMC11273692; doi:10.3390/antibiotics13070680)
Supplement: Supplementary file 1 [file antibiotics-13-00680-s001.zip › antibiotics-3058586-supplementary.pdf]

antibiotics

MDPI

Supplementary Materials

Synergistic Antimicrobial Effects of Phage vB_AbaSi_W9 and Antibiotics against *Acinetobacter baumannii* Infection

Yoon-Jung Choi, Shukho Kim, Minsang Shin and Jungmin Kim *

Department of Microbiology, School of Medicine, Kyungpook National University, Daegu 37224, Republic of Korea; yjchoi8727@knu.ac.kr (Y.-J.C.); shukhokim@knu.ac.kr (S.K.); shinms@knu.ac.kr (M.S.)
* Correspondence: minkim@knu.ac.kr; Tel.: +82-53-420-4845, Fax.: +82-53-427-5664

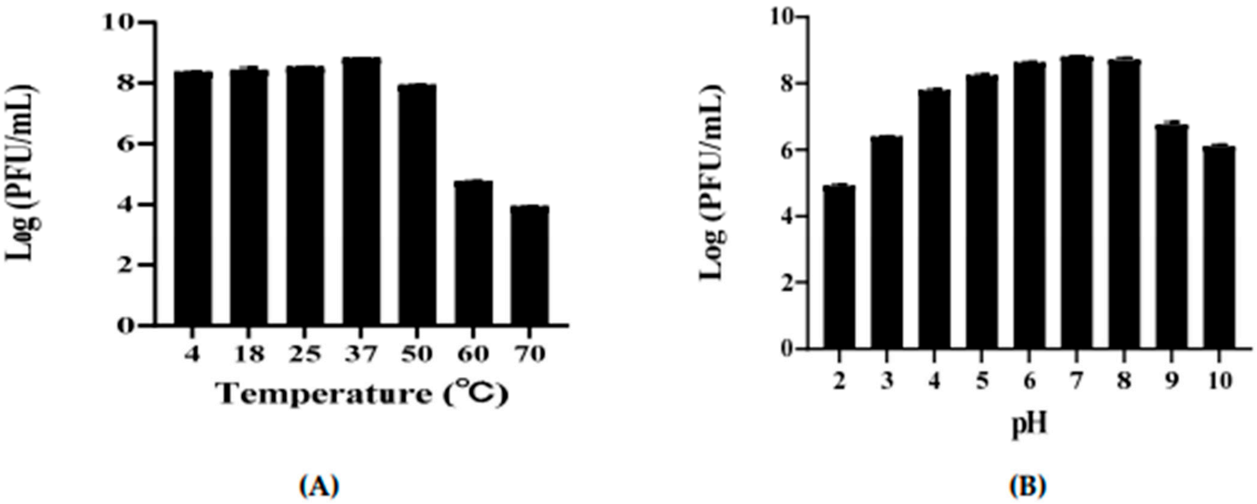

Figure S1. Temperature and pH stability of phage vB_AbaSi_W9. Each data point represents the mean of three independent experiments, with standard deviations indicated by vertical lines. Results are expressed as Log PFU/mL. (**A**) Temperature stability of phage. (**B**) pH stability of phage.
